# Microscopic retrograde great saphenous vein supercharged anastomosis to address propeller flap congestion in lower limb defect reconstruction: A case report

**DOI:** 10.1016/j.ijscr.2025.111532

**Published:** 2025-06-20

**Authors:** Ali Yavari, Hesam Amini, Pedram Akhlaghi

**Affiliations:** a Plastic and Reconstructive surgery Department, Medicine school, Tehran University of Medical Sciences, Tehran, Iran; b Department of Thoracic surgery, Imam Khomeini Hospital Complex, Tehran University of Medical Sciences, Tehran, Iran; c Faculty of Biomedical Engineering, Amirkabir University of Technology, Tehran, Iran

**Keywords:** Propeller flap, Retrograde venous supercharge, Microscopic anastomosis, Lower limb congestion

## Abstract

**Introduction:**

Reconstructing defects in the lower limb can be challenging when local flaps cannot be used. Free flaps are a good alternative, but they take more time. Free flaps may also fail in inflamed areas because veins can be damaged by inflammation.

**Case presentation:**

We report a 35-year-old man with a severe leg and ankle injury. We treated his defect with a propeller flap that included the great saphenous vein, clipped at its proximal end. After moving the flap over the wound, the clipped end of the saphenous vein was directed toward the ankle. We then connected this clipped end to a deep vein in the ankle by a retrograde end-to-end anastomosis. This was done to prevent venous congestion and improve venous drainage.

**Discussion:**

Propeller flaps based on perforator vessels can substitute for free flaps when other local flaps are unavailable. However, propeller flaps often have venous congestion as a complication. Performing a venous supercharging anastomosis, even in a retrograde manner, can help reduce this congestion.

**Conclusion:**

Propeller flaps are an excellent option for lower limb reconstruction. A microscopic retrograde anastomosis of the great saphenous vein can effectively prevent venous congestion.

## Highlights

• Reconstructing defects in the lower limb can be difficult.
• Local flaps may not be viable, necessitating alternative options.
• Free Flaps are effective, but can be time-consuming and unsuitable for inflamed tissues.
• Propeller Flaps offer a practical alternative to free flaps, but venous congestion is a frequent complication.
• Venous supercharging can alleviate venous Congestion.

## 1 Introduction

Reconstructing lower limb defects can be particularly difficult because of blood supply issues. Perforator flaps, such as propeller flaps, are an effective alternative to free flaps when local flaps are not available [1,2]. These flaps take less surgical time and provide tissue similar in color and texture to the defect area. They also avoid donor site complications [1,2].

Venous congestion is the most common complication of propeller flaps. A practical way to reduce congestion is venous supercharging. Surgeons often include the saphenous vein (great or lesser) in the flap to improve drainage, using either a retrograde or antegrade connection [3]. In this report, we present a technique for supercharging flaps by microscopically anastomosing the great saphenous vein to a deep vein at the defect site. This connection allows the flap to drain in a retrograde manner. This case report follows the 2025 SCARE guidelines [12].

## 2 Case presentation

A 35-year-old man was injured in a car accident. He had fractures of the tibia and fibula and underwent open reduction and internal fixation (ORIF) of the ankle. Twenty-six days later, he was referred to the plastic surgery department. On exam, the ankle joint and distal leg bones, including the orthopedic plate, were exposed. Because of the lateral malleolus incision, we could not use a reverse saphenous or lateral supramalleolar flap. Instead, we used a fasciocutaneous propeller flap based on a medial posterior tibial artery perforator to cover the 11 × 18 cm defect.

**Fig. 1:**
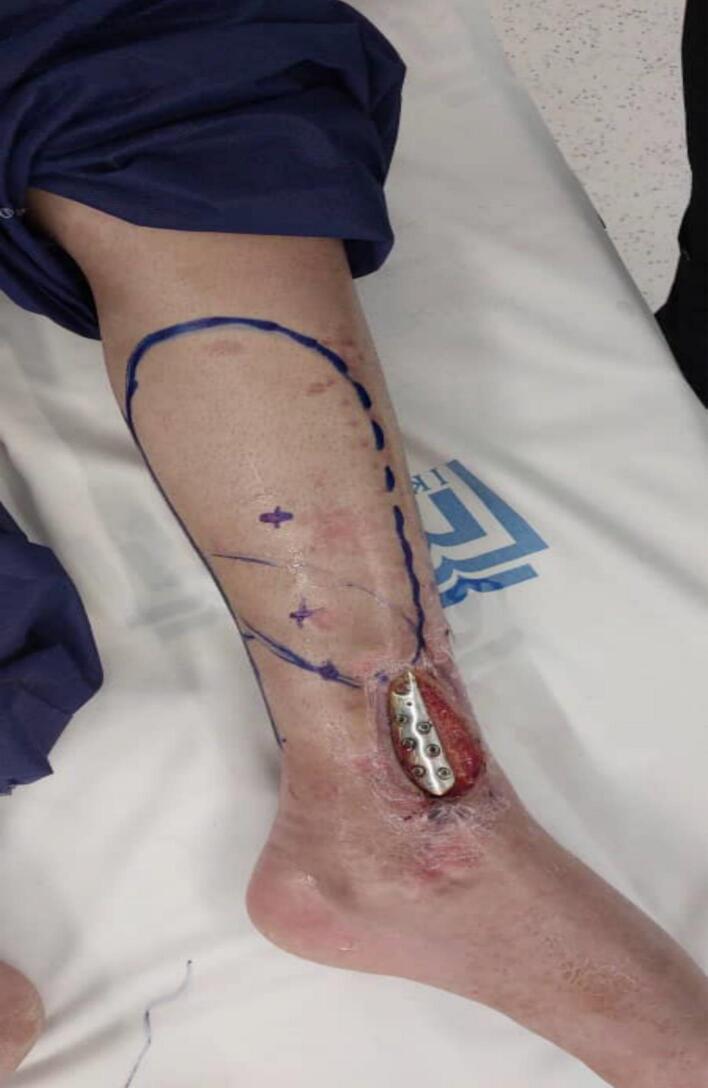
The defect of ankle.

**Fig. 2:**
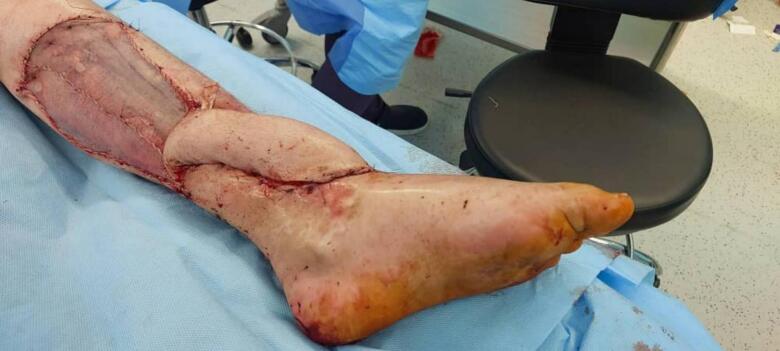
The defect covered with Propeller Flap.

**Fig. 3:**
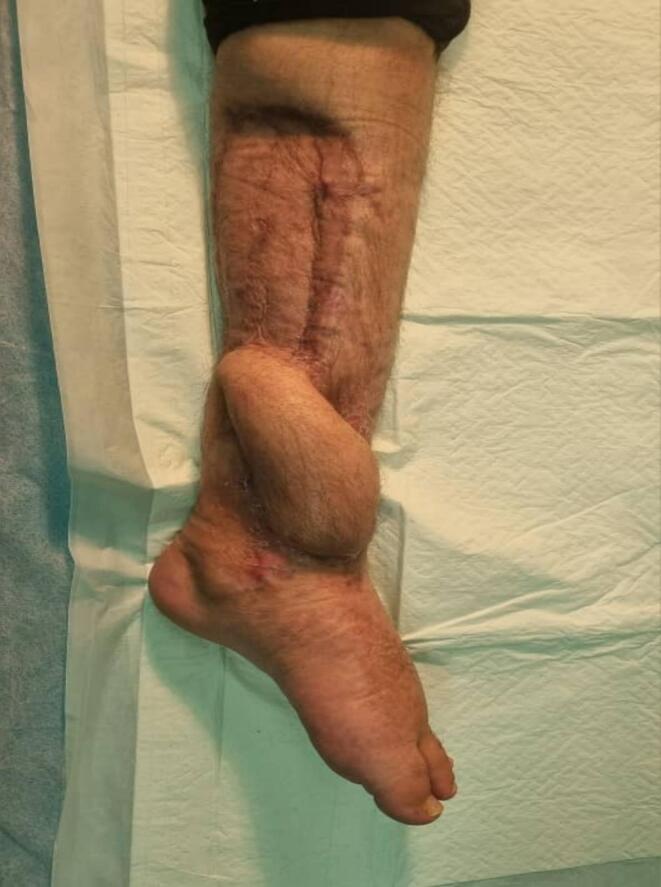
The limb at 1 month follow-up.

Before surgery, his vital signs were stable (blood pressure 120/80 mmHg, heart rate 65 bpm, respiratory rate 21 breaths/min, temperature 37 °C) and oxygen saturation was 97 %. Laboratory tests (CBC, PT, PTT, INR, ESR, CRP) were all normal. Doppler ultrasound detected two intact posterior tibial artery perforators in the medial leg. We chose the perforator closest to the defect for the flap (Fig. 1, Fig. 2, Fig. 3).

Under general anesthesia, we marked the flap over the medial leg, including the identified perforators. We made an incision and carefully dissected the flap. We included the great saphenous vein in the flap while preserving the perforator and surrounding tissues. The great saphenous vein was clipped at its proximal end.

After elevating and rotating the flap over the defect, the clipped end of the saphenous vein was positioned toward the ankle. We made an incision at the ankle and lower leg to identify a deep recipient vein. Initially, the saphenous vein was larger than the recipient vein, so we closed half of its lumen with 9–0 prolene sutures. Under 2.5× magnification, we then performed a microscopic retrograde end-to-end anastomosis connecting the proximal clipped end of the saphenous vein to the deep leg vein. We used 9–0 polypropylene sutures for the anastomosis. We secured the flap over the defect with a tension-free closure. The donor site was covered with a split-thickness skin graft. The operation lasted approximately two and a half hours.

Postoperatively, the wound was dressed and the patient was moved to the ward. He received IV antibiotics (cephazolin 1 g every 6 h) and IV heparin (1000 units/h) for five days. We examined the flap every 6 h for blood flow and congestion. On the sixth day, we removed the dressing on the donor site. The flap had normal color and capillary refill. The patient recovered without complications and was discharged with 80 mg of aspirin daily and pantoprazole. He returned to the clinic weekly for two months. At the 2-month follow-up, the wound had completely healed.

## 3 Discussion

When planning reconstruction of a lower leg or ankle defect, surgeons must consider the size and depth of the wound, the condition of surrounding tissue (especially in fractures), the status of blood vessels for free flap transfer, and the availability of nearby tissue [1]. Propeller flaps have become popular in recent years [4]. D'Arpa et al. noted that propeller flaps have a reliable vascular pedicle and allow great freedom of design. They can cover difficult wounds with local tissue and cause little donor site morbidity. These flaps also allow one-stage reconstruction of defects that might otherwise need multiple operations. However, using a propeller flap requires careful patient selection, planning, and surgical technique. If potential problems are prevented and recognized early, the complication rate can be kept low [4].

For defects of the distal leg and ankle, one might consider reverse sural or lateral supramalleolar flaps. However, in our patient the lateral incision made these options unsafe. Yavari et al. reported successful reconstruction of a severe knee defect using a propeller flap that included the great saphenous vein [5]. They performed a microscopic retrograde anastomosis between the clipped proximal end of the saphenous vein and a deep vein in the leg. This connection overcame venous congestion despite the retrograde flow [5]. Atmodiwirjo et al. described a similar case of an Achilles tendon area defect. They used a peroneal artery perforator propeller flap with a microscopic venous anastomosis to prevent congestion [6]. Their case is similar to ours, except we used a flap based on a posterior tibial artery perforator.

Chaput et al. compared standard propeller flaps (PPF) with venous-supercharged propeller flaps (vsPPF) [7]. They found that vsPPF required longer surgery time but had much lower rates of venous congestion (6.7 % vs 36.7 %) [7]. They concluded that venous-supercharged propeller flaps are a reliable alternative to standard propeller flaps [7]. Mishra et al. reported a case like ours using a medial leg perforator flap [8]. They included the great saphenous vein in their flap but did not perform a venous anastomosis, and still achieved a successful result [8].

Humnekar et al. compared propeller flaps and free flaps for ankle and distal leg reconstruction They found that propeller flaps had better outcomes: they required a smaller flap, had shorter surgery time, and led to shorter hospital stays [9]. In contrast, Ota et al. found that propeller flaps had higher complication rates than free flaps in traumatic cases [10]. These opposite findings suggest that outcomes can vary depending on the surgeon's experience and case selection.

Goud et al. used a distally based lateral supramalleolar flap to cover an ankle defect and reported good results [11]. They found this flap effective for moderate-sized defects of the distal foot and ankle, with few complications [11]. The lateral supramalleolar flap is based on a distal perforator of the peroneal artery [11]. In our case, previous lateral incisions likely injured these perforators, making this flap risky. Moreover, the lateral supramalleolar flap works best in patients with healthy skin and is less useful in cases with recurrent wounds or trauma. In such challenging cases, regional perforator flaps like propeller flaps are more appropriate. These flaps use local tissues, cause less donor site damage, and require fewer microsurgical connections. They represent a modern advance in reconstruction. Although learning these techniques can be difficult at first, they allow surgeons to address severe limb injuries effectively.

Venous supercharging itself is not new, but our retrograde approach is an innovation. This method appears safe even in complex lower limb defects. It is efficient because it requires only one venous anastomosis instead of two (as would be needed to connect two veins), which saves operative time. However, challenges include the size mismatch between the veins, which demands microsurgical expertise. Also, in traumatic flaps the local perforators may be damaged, so finding a suitable perforator near the defect can be difficult and may require using a larger flap.

## 4 Conclusion

This case illustrates the successful use of a medial leg perforator flap to repair a complex lower leg injury after trauma. It emphasizes important surgical steps and highlights the role of propeller perforator flaps in lower extremity reconstruction. It also shows that a microscopic retrograde anastomosis of the great saphenous vein can reduce venous congestion in such flaps. Future studies should explore the versatility of this technique in other cases.

## Author contribution

AY conceived the study. HA contributed to the case collection, discussion, literature review, and first manuscript draft. HA and AY provided critical revisions. PA contributed in writing, editing and reviewing. All authors contributed to the article and approved the submitted version.

## Consent for publication

Written informed consent was obtained from the patient for publication of this case report and images. A copy of the consent is available on request.

## Ethical approval

Ethical approve was obtained from Institutional Review Committee of Tehran University of Medical Sciences (IR.TUMS.IKHC.REC.1403.458). Written and oral consent was obtained from patient.

## Guarantor

Hesam Amini

## Funding

No source of funding.

## Declaration of competing interest

The authors declare no conflicts of interest regarding this case report.
